# Methanol diffusion and dynamics in zeolite H-ZSM-5 probed by quasi-elastic neutron scattering and classical molecular dynamics simulations

**DOI:** 10.1098/rsta.2022.0335

**Published:** 2023-10-30

**Authors:** Santhosh K. Matam, Ian P. Silverwood, Lotfi Boudjema, Alexander J. O'Malley, C. Richard A. Catlow

**Affiliations:** ^1^ UK Catalysis Hub, Research Complex at Harwell, Science and Technology Facilities Council, Rutherford Appleton Laboratory, Didcot OX11 0FA, UK; ^2^ Cardiff Catalysis Institute, School of Chemistry, Cardiff University, Cardiff CF10 3AT, UK; ^3^ ISIS Pulsed Neutron and Muon Facility, Science and Technology Facilities Council, Rutherford Appleton Laboratory, Didcot OX11 0QX, UK; ^4^ Department of Chemistry, University College London, 20 Gordon Street, London WC1E 6BT, UK; ^5^ ICGM, Université de Montpellier, CNRS, ENSCM, Montpellier, France; ^6^ Institute for Sustainability, Department of Chemistry, University of Bath, Bath BA2 7AY, UK

**Keywords:** H-ZSM-5, Si/Al ratio, *Brønsted* acidity, methanol, QENS, diffusion and dynamics

## Abstract

Zeolite ZSM-5 is a key catalyst in commercially relevant processes including the widely studied methanol to hydrocarbon reaction, and molecular diffusion in zeolite pores is known to be a crucial factor in controlling catalytic reactions. Here, we present critical analyses of recent quasi-elastic neutron scattering (QENS) data and complementary molecular dynamics (MD) simulations. The QENS experiments show that the nature of methanol diffusion dynamics in ZSM-5 pores is dependent both on the Si/Al ratio (11, 25, 36, 40 and 140), which determines the *Brønsted* acid site density of the zeolite, and that the nature of the type of motion observed may vary qualitatively over a relatively small temperature range. At 373 K, on increasing the ratio from 11 to 140, the observed mobile methanol fraction increases and the nature of methanol dynamics changes from rotational (in ZSM-5 with Si/Al of 11) to translational diffusion. The latter is either confined localized diffusion within a pore in zeolites with ratios up to 40 or non-localized, longer-range diffusion in zeolite samples with the ratio of 140. The complementary MD simulations conducted over long time scales (1 ns), which are longer than those measured in the present study by QENS (≈1–440 ps), at 373 K predict the occurrence of long-range translational diffusion of methanol in ZSM-5, independent of the Si/Al ratios (15, 47, 95, 191 and siliceous MFI). The rate of diffusion increases slightly by increasing the ratio from 15 to 95 and thereafter does not depend on zeolite composition. Discrepancies in the observed mobile methanol fraction between the MD simulations (100% methanol mobility in ZSM-5 pores across all Si/Al ratios) and QENS experiments (for example, ≈80% immobile methanol in ZSM-5 with Si/Al of 11) are attributed to the differences in time resolutions of the techniques. This perspective provides comprehensive information on the effect of acid site density on methanol dynamics in ZSM-5 pores and highlights the complementarity of QENS and MD, and their advantages and limitations.

This article is part of the theme issue ‘Exploring the length scales, timescales and chemistry of challenging materials (Part 2)’.

## 1. Introduction

Zeolite ZSM-5 plays a crucial role in many commercial petrochemical and environmental processes such as methanol to hydrocarbons (MTH) due to its unique porous architecture and catalytic properties [1–4]. The former influences the product selectivity, while the latter mainly originates from its *Brønsted* acidity, which depends on the Si/Al ratio of the zeolite [2–4]. The MTH process enables replacement of conventional fossil fuels such as coal, crude oil and natural gas with carbon neutral renewable methanol feedstock to produce gasoline, olefins and aromatics, and therefore can play a key role in achieving the net zero carbon emissions target by 2050 [5]. Consequently, there is a growing interest in unravelling the fundamental reaction and deactivation mechanisms [4,6–11]. A consensus on the role of *Brønsted* acidity in the first C–C bond formation mechanism from C1 methanol molecule has emerged [4,6–10]. However, studies on the initial stage of the MTH process involving adsorption, diffusion and reaction of molecules at the *Brønsted* acid site are very limited [12–16]; and as diffusion rates often determine the reaction rates, the understanding of diffusion dynamics is crucial for designing and tailoring further the acidity and porous architecture for the development of an efficient catalytic process [17]. In this perspective, we discuss in detail the effect of acid site density, which in turn depends on the Si/Al ratio of ZSM-5, on the methanol diffusion dynamics and show how the combination of experimental neutron scattering studies with molecular simulations can reveal the complexity of the dynamical behaviour of sorbed methanol in the pores of zeolite ZSM-5.

## 2. Probing molecular diffusion in zeolites

In recent years, methanol diffusion dynamics in ZSM-5 pores have been studied by quasi-elastic neutron scattering (QENS) [18–20], which provides an insight into methanol behaviour on the scale of nano- and picoseconds that is not covered by techniques such as pulsed-field gradient nuclear magnetic resonance or the classical sorption uptake method [18,21]. Moreover, the QENS data can be complemented by molecular dynamics (MD) simulations that essentially probe a similar time and length scale as QENS, although the time resolution of the techniques can vary depending on the dynamics being simulated by MD or on the QENS instruments being employed. Methanol diffusion in ZSM-5 with varied Si/Al (15, 47, 95 and 191) has been studied by MD simulations [22,23]; however, no such systematic experimental studies are reported previously. Most of the QENS studies employed ZSM-5 with a Si/Al ratio of ≈30 and conducted temperature-dependent diffusion dynamics between 298 and 400 K [12–16]. These studies, however, led to inconsistent conclusions, which could be attributed to the differing resolutions and configurations of the instruments employed [12,16], and also the differing loadings and sample characteristics. While a study reports long-range translational diffusion along the ZSM-5 channels with a self-diffusion coefficient (*D*_s_) of ≈10^−11^ m^2^ s^−1^ at 300 K [12], the others conclude either no methanol mobility [13–15] or no long-range translational methanol diffusion in ZSM-5 at the same temperature range [16,24,25]. The former study employed the relatively higher resolution IN10 instrument at Institut Laue-Langevin (ILL), Grenoble, while the latter used the OSIRIS or IRIS instrument at the ISIS Pulsed Neutron and Muon Source (ISIS), Harwell. Although the higher resolution of IN10 allows measurement of slower motions (≈100–1000 ps), OSIRIS and IRIS are well-suited to study sorbates in like zeolites whose diffusional dynamics are on the timescale of ≈1–440 ps. Higher temperatures, which may also influence the diffusion mechanism [24,25] or lead to reaction, may be needed for the detection of the elastic peak broadening, discussed below, of slower diffusion rates. Therefore, the comparisons of data from different instruments across a broad temperature range should be treated with caution. Here, we focus on data acquired using the OSIRIS (time resolution: ≈1–100 ps) and IRIS (time resolution: ≈3–440 ps) instruments (at the ISIS Neutron and Muon Source, Harwell) with comparable time and length scale resolutions. The experimental data are compared with the results of MD simulations (time resolution: ≈1 ns) [22,23]. Critical analyses of the present and previous experimental and computational data reveal that the acid site density of the zeolite is a crucial factor in determining the nature of methanol dynamics in H-ZSM-5 pores.

## 3. Techniques

### (a) Quasi-elastic neutron scattering

QENS measures the scattering function *S,* as a function of energy transfer *ω* and momentum transfer vector **Q**: *S*(**Q**,*ω*). Although the direction of the vector can aid interpretation in single crystalline samples, only the magnitude can be obtained for powders. Energy transfer occurs when neutrons scatter from atoms in motion and from this the kinetics can be determined. As there is a directional component to the momentum transfer, the geometry of this motion can also be inferred.

The scattering function is linked to the van Hove correlation function, *G*, through the double time and space Fourier transform [26]. *G*(**r**,*t*) describes the probability of finding an atom at a given radius and time from the origin and is inversely proportional to *S*(**Q**,*ω*). The correlation function can be subdivided into the self- and distinct-partial correlation functions, which represent the probability of finding the same atom, or a distinct atom at a given radius and time. In **Q**,*ω* space, the incoherent scattering represents the self-correlation function and the coherent scattering is related to the distinct-correlation function. While the interpretation is relatively easy for monatomic liquids, heterogeneous and multi-phase systems rapidly complicate the picture.

The strength of the scattering interaction between a neutron and an atomic nucleus is experimentally measured due to its apparently random nature. It is isotopically dependent and has both a coherent and an incoherent contribution. These components vary individually. As the entire scattering function is experimentally measured, separation of the coherent and incoherent scattering is of primary importance. Since the incoherent scattering cross section of ^1^H is much larger than that for most atoms, the approximation is often made that all the incoherent scattering in a system results from hydrogen. More concerning, however, is the **Q**-dependent coherent elastic scattering. This can result in strong Bragg peaks that interfere with the characterization of the incoherent scattering.

Localized and non-localized long-range translational dynamics of hydrogenous molecules such as methanol can be probed by QENS as described in detail in [12,18]. Localized motions such as isotropic rotation, uniaxial rotation or methyl jump rotation and confined translational motions can be studied by the elastic incoherent structure factor (EISF), which is the proportion of total scattered intensity and is elastic. The EISF is given by equation (3.1).

3.1 $$A_{0}(Q)=\frac{I_{\text{elastic}}(Q)}{I_{\text{elastic}}(Q)+I_{\text{inelastic}}(Q)}.$$

**Figure 1.**
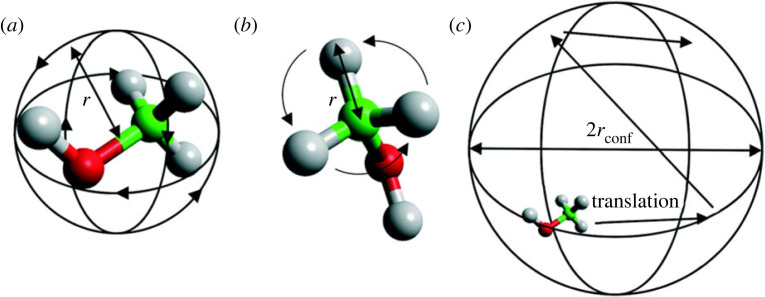
Schematic of localized methanol motions: (*a*) isotropic rotation, (*b*) either methyl jump rotation or uniaxial rotation and (*c*) translational diffusion confined to a sphere. Adapted from [27]—published by The Royal Society of Chemistry.

The geometries of localized motions are depicted in figure 1.

Detailed description of localized models and their theoretical treatment can be found elsewhere [12,16,18,24,27]. Briefly, isotropic rotation is characterized by a molecule whose reorientation takes place through a series of small angle, random rotations so that no most probable orientation exists on a time average as depicted in figure 1*a*. The scattering law for this form of rotation has an EISF $\left(A_{0}(Q)\right)$ [28]:

3.2 $$A_{0}(Q)=\;j_{0\;}^{2}(Qr),$$

where *r* is the radius of rotation, and *j*_0_ is the 0th order spherical Bessel function given as

3.3 $$j_{0}(Qr)=\frac{\sin(Qr)}{\left(Qr\right)}.$$

The average radius of rotation of the four methanol protons as calculated from the centre of mass is 1.48 Å.

The methyl jump rotation model describes a methanol molecule which is fixed through adsorption to the zeolite surface such that the only motion observable is that of rotating methyl groups. Methyl jump rotation occurs between three equidistant sites on a circle with a radius (*r*) as depicted in figure 1*b* and the corresponding EISF is

3.4 $$A_{0}(Q)=\frac{1}{3}\left[1+2j_{0}\left(Qr\sqrt{3}\right)\right],$$

where *r* is the radius of the circle on which the jumps take place, in this case 1.02 Å.

The model depicted in figure 1*b* may also be described by the uniaxial rotation model [29] if the methyl protons undergo continuous rotation around a circle. For this, a jump rotation over *N* sites around a circle, similar to the 3-site model discussed above (equation (3.4)), with a sufficiently large *N* (greater than 7). Then the jump rotation over *N* sites may be used to approximate continuous rotation, equation (3.5).

3.5 $$A_{0}(Q)=\frac{1}{N}\overset{N}{\underset{n=1}{\sum }}j_{0}\left[2Qr_{\text{u}}\sin\left(\frac{n\pi }{N}\right)\right].$$

Figure 1*c* depicts the localized translational diffusion of methanol within a spherical volume such as a pore or cage with a radius of *r*_conf_. The scattering model is based on the general assumption of a particle diffusing in a potential field of spherical symmetry, where the potential is low inside the sphere's volume but infinite outside of it, equation (3.6):

3.6 $$A_{0}(Q)={\left[\frac{3j_{1}(Qr_{\text{conf}})}{Qr_{\text{conf}}}\right]}^{2},$$

where *j*_1_ is the spherical Bessel function of the first kind, order 1.

Volino and Dianoux (VD) [30] developed a model to describe a scattering molecule undergoing translational diffusion within a confined spherical volume of radius *r*_conf._ as shown in figure 1*c*. Finally, the theoretical background of long-range translational diffusion of methanol is briefly discussed here and a detailed description can be found elsewhere [12]. Translational motions of the hydrogenous molecules such as methanol are often assumed to follow Fick's law at lower *Q*, corresponding to macroscopic length scales. The van Hove self-correlation function allows us to describe the incoherent scattering function [12,18] and then the HWHM of the Lorentzian function has a linear relationship with the *D*_s_ and the square of momentum transfer (*Q*^2^), as shown by equation (3.7).

3.7 $$\Delta \omega {\left(Q\right)}_{\text{incoh}}=D_{\text{s}}Q^{2}.$$

This relationship is strictly valid only for ideal Fickian diffusion. Deviation from this behaviour, at higher *Q* values (i.e. on the molecular level), is common for heterogenous catalytic systems such as methanol diffusion in ZSM-5 involving frequent interactions between methanol molecules and zeolite framework. These interactions encompass elementary diffusion steps for which jump diffusion models are developed [12,18], which assume that diffusing molecules jump from one site to another which are separated by a certain distance (*d*). While the jump is rapid, molecules rest on a particular site for a given resident time (*τ*). Among the different models, Chudley–Elliot (CE) formalism [31], assuming constant jump distance, generally fits the data satisfactorily (equation (3.8)).

3.8 $$\Delta \omega (Q)=\frac{1}{\tau }\left(1-\frac{\sin(Qd)}{Qd}\right).$$

Fitting this function provides the jump distance (*d*) and residence time (*τ*). *D*_s_ can be determined using equation (3.9), which is valid for the CE model [12,18].

3.9 $$D_{\text{s}}=\frac{d^{2}}{6\tau }.$$

### (b) Classical molecular dynamics simulations

The time and length scales associated with dynamical methanol behaviour in ZSM-5 have typically required force field-based (so-called ‘classical’) MD simulations. Though these are of a lower level of theory than quantum mechanical simulations, detailed qualitative and quantitative information may be obtained about dynamics from the rotational motions on the scale of 1–100 ps all the way through to the longer-range diffusion, which takes place on the scale of several nanoseconds.

The force fields upon which these simulations are based are relatively simple mathematical expressions of how the potential energy changes as a function of interatomic distance. Coulomb potentials describe electrostatic interactions; van der Waals interactions are typically described by Buckingham or Lennard-Jones potentials, and harmonic stretching, bending or torsional potentials describe intramolecular bonded interactions. The gradient of these potentials is then used to calculate the forces on each atom, after which Newton's laws of motion may be integrated to model the motion of atoms, and the evolution of the system as time passes.

We point the reader to reviews on the use of modelling techniques in zeolite science to study adsorption, diffusion and reaction by Van Speybroeck *et al*. [4] and Smit & Maesen [32], which inform on the multitude of advances in computational methods and their contributions to studying zeolite-mediated processes. One significant development in the last 10 years relevant to this perspective article is the routine use of zeolite frameworks, which are modelled to include framework flexibility—where the vast majority of modelling studies of diffusion in zeolites before this would employ a rigid framework approximation to save on computational expense, which can potentially have significant effects on the dynamical behaviours of adsorbed molecules [33].

The long-range diffusion of methanol in ZSM-5 may be quantified through calculating the diffusion coefficient from atomic displacements recorded from the MD simulation, using the Einstein relationship:

3.10 $$D_{\text{s}}=\frac{1}{6}\underset{t\to \infty }{\lim}\left(\frac{\text{d}}{\text{d}t}\right)\langle {\left(r(t)-r(0)\right)}^{2}\rangle ,$$

where the term in braces is the ensemble average of the MSD of the methanol molecule.

In terms of directly reproducing QENS observables, the dynamic structure factor (*S* (*Q*, *ω*)) is related to the single-particle temporal and spatial correlation functions; these give rise to the intermediate scattering function (*F*_s_(*Q*, *t*)), which is related to the dynamic structure factor by a Fourier transformation in the time domain:

3.11 $$S(Q,\omega )=\frac{1}{\pi }\int F_{\text{s}}(Q,t){\text{e}}^{\left(-\text{i}\omega t\right)}\text{d}t.$$

*F*_s_(*Q*, *t*) may be derived from the atomic displacements from MD simulations, as shown in equation (3.12). Here, *N* is the number of, and *r_i_* is the position of each hydrogen atom *i*. If rotation is being probed, we may use the position of each hydrogen atom relative to the centre of mass of the molecule.

3.12 $$F_{\text{s}}(Q,t)=\frac{1}{N}\overset{N}{\underset{i=1}{\sum }}\left\langle \frac{\sin(|Q||r_{i}(t+t_{0})-r_{i}(t_{0})|)}{\left(|Q||r_{i}(t+t_{0})-r_{i}(t_{0})|\right)}\right\rangle .$$

We may fit the simulation-derived *F*_s_(*Q*, *t*) with one or more exponential functions, each representing a different motion (as in equation (3.13)). The pre-exponential factor *C* denotes the weighting factor (how much each exponential, and therefore the motion it represents, contributes to the total *F*_s_(*Q*, *t*)). The decay constant *Γ* is the rate of the motion and can be considered the same as the half width half maxima (HWHM) of the Lorentzian used to fit the *S*(*Q*, *ω*). The constant *B*(*Q*) represents the EISF, which may also act as a point of direct comparison.

3.13 $$F_{\text{s}}(Q,t)=C_{1}Q{\text{e}}^{-\Gamma_{1}t}+C_{2}Q{\text{e}}^{-\Gamma_{2}t}+B(Q).$$

A detailed review of the direct reproduction of QENS observables from MD simulations and its application in catalysis and energy materials may be found in [34].

## 4. Experimental details

The experimental details are similar to those reported elsewhere [16,24,25]. Briefly, the NH_4_-ZSM-5 with Sil/Al ratios of 11 (product code: CBV 2314), 40 (product code: CBV 8014) and 140 (product code: CBV 28014) are purchased from Zeolyst International, Inc. The zeolites were calcined in air at 500°C for 24 h to obtain H-ZSM-5. Methanol loading was conducted at room temperature by flowing dry N_2_ through a methanol saturator. The loading was conducted until there was no methanol uptake by the zeolites, as determined gravimetrically. At this saturation level the loading resulted in four methanol molecules per *Brønsted* acid site. QENS experiments were conducted using the time-of-flight backscattering OSIRIS (time resolution: ≈1–100 ps) or IRIS (time resolution: ≈3–440 ps) spectrometers at the ISIS, Harwell [12,24,25]. The zeolites with and without methanol loading were analysed. A resolution measurement of each unloaded (i.e. without methanol) zeolite was obtained at a base temperature of around 10 K. QENS measurements on both zeolites with and without the methanol load were conducted at 298 and 373 K. Pyrolytic graphite 002 analyser crystals were used [16,24,25]. The QENS data collected without methanol loading were subtracted from the data obtained with the loaded zeolite, which leaves the scattering signal only from the methanol.

## 5. Methanol diffusion dynamics as a function of Si/Al ratio of H-ZSM-5

The effect of Si/Al ratio of H-ZSM-5 on the methanol diffusion dynamics is studied by QENS using zeolites with varied Si/Al ratios, including those previously reported (table 1).

**Table 1.** ZSM-5 with varied Si/Al ratios are considered for this study. Methanol loading at saturation levels.

s. no.	Si/Al	reference
1	11	present study
2	25	[24]
3	30	[12]
4	36	[23]
5	40	present study
6	140	present study

Based on previously reported QENS data, it is evident that the type of motion observed can change significantly in a relatively small temperature window. For example, H-ZSM-5 with an Si/Al ratio of 25 has been observed to show isotropic rotation of methanol at 298 K and then translational diffusion of methanol confined to a pore volume of the zeolite at 373 K [25]. The temperature-dependent behaviour also holds true for H-ZSM-5 with an Si/Al ratio of 36 [24], i.e. the switch from rotational dynamics to translational upon increasing the measurement temperature from 298 to 373 K. Therefore, QENS studies conducted at a constant temperature of 373 K are considered below (§5a) to eliminate the temperature-dependent methanol dynamics in zeolite pores and to probe explicitly the effect of the ratio on the dynamics.

### (a) Methanol diffusion dynamics at 373 K

#### (i) H-ZSM-5 with Si/Al of 11

**Figure 2.**
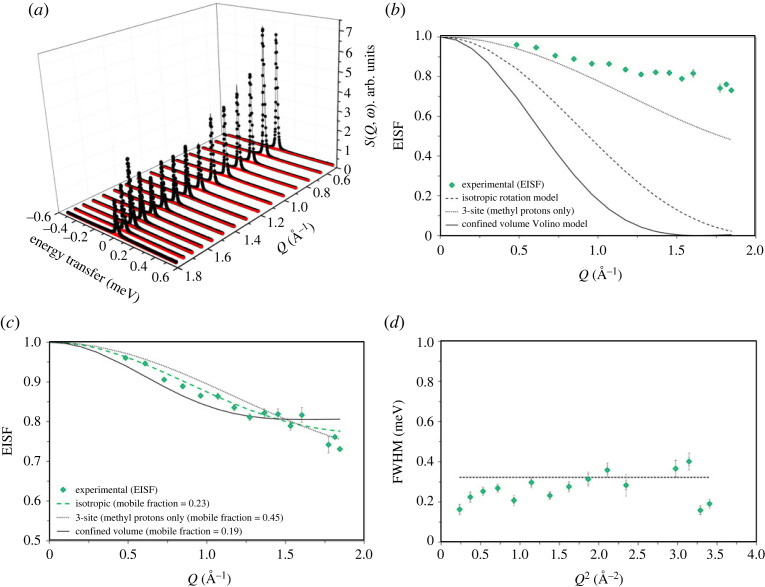
(*a*) QENS spectra of methanol loaded H-ZSM-5 with Si/Al of 11 at 373 K. Experimental data (filled circles), total fit (intense feature shown as a solid black line in the colour version online) and the Lorentzian function (broader, lower intensity function shown in red in the colour version). (*b*) The corresponding experimental elastic incoherent structure factor (EISF) data (diamond symbols) are compared with different theoretical models that are discussed in detail in [12,16,25]. (*c*) The 3-site and uniaxial rotation models appear identical and hence the latter model is not shown in (*b*) and (*c*). Experimental EISF data are modelled by including a fraction of immobile methanol in different theoretical models. Mobile methanol fraction is shown in the parenthesis of (*c*) and confined volume radius of 2.75 Å is considered. (*d*) The FWHM of the Lorentzian function is plotted as a function of *Q*^2^. The *Q* values at 1.66 and 1.72 Å^−1^ are excluded due to potential contribution from Bragg peaks. The data were collected on an IRIS instrument and were grouped over 17 detectors.

The H-ZSM-5 with the highest acid site density studied in the series has an Si/Al of 11, denoted as ZSM-5-11, where we present QENS data, which are depicted in figure 2. The experimental QENS data fit adequately to the combination of a delta function and a single Lorentzian function convoluted with the resolution data collected at 10 K and a linear background. The delta function represents elastic scattering and the Lorentzian function, the inelastic. The scattering function, *S* (*Q*,*ω*), is plotted together with the fitted Lorentzian function in figure 2*a*. It is evident that the total intensity of the QENS spectrum is due to a dominant contribution of elastic scattering, especially at low *Q* (less than 1.1 Å^−1^) values. The intensity of the elastic peak decreases with increasing *Q*, while the fraction of total intensity from the Lorentzian function increases, which is analysed by the EISF—the ratio between the elastic peak intensity and total peak intensity as calculated from the values derived by peak fitting. This ratio provides information on the localized methanol dynamics, as described in §3a [12,18,24,25]. The experimental EISF is compared with different theoretical models in figure 2*b*. The EISF does not fit to any of the pure models that assume complete mobility of methanol in ZSM-5 pores, suggesting that the methanol is not completely mobile in ZSM-5-11 pores. Thus, the experimental EISF is described by the effective EISF models [24,25] that include a fraction of immobile methanol (figure 2*c*). It is evident that the experimental EISF now adequately fits to the isotropic rotation of the methanol model with a mobile fraction of 23%, whereas the models 3-site methyl jump rotation and translational diffusion of methanol in a confined spherical volume (with an assumed radius of 2.75 Å), corresponding to the zeolite pore size, do not adequately fit the experimental EISF data. The EISF fit is further analysed by the full width at half maxima (FWHM) of the Lorentzian function as a function of Q^2^ (figure 2*d*). The FWHM appears to be *Q*^2^ independent, which is characteristic of localized dynamics such as isotropic rotation of methanol, corroborating the EISF fitting. The rotational diffusion coefficient (*D*_R_) can be derived from FWHM as described elsewhere [12,16] and it gives rise to an isotropic methanol *D*_R_ of 1.2 × 10^11^ s^−1^. Upon increasing the Si/Al ratio of ZSM-5 from 11 to 25 [25], 36 [24] and 40 (present study), the methanol diffusion dynamics switch from rotational to translational, as discussed below.

#### (ii) H-ZSM-5 with Si/Al of 40

**Figure 3.**
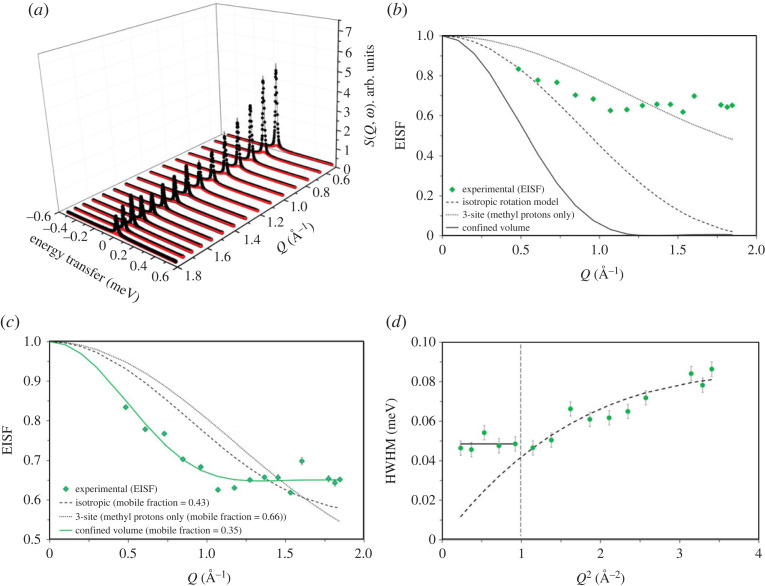
(*a*) QENS spectra of methanol loaded H-ZSM-5 with Si/Al of 40 at 373 K. Experimental data (filled circles), total fit (intense feature shown as a solid black line in the colour version online) and the Lorentzian function (broader, lower intensity function shown in red in the colour version). (*b*) The corresponding EISF (diamond symbols) are compared with different theoretical models [12,16]. The 3-site and uniaxial rotation models appear identical and hence the latter model is not shown in (*b*) and (*c*). (*c*) Experimental EISF data are modelled by including a fraction of immobile methanol in different theoretical models. Mobile methanol fraction is shown in the parenthesis of (*c*) and the estimated confined volume radius is 3.36 Å. (*d*) The *Q* dependence of the HWHM of the Lorentzian function fits well with the Volino–Dianoux (VD) confined diffusion model (*Q*^2^ < 1 Å^−2^; indicated by the dashed black vertical line in (*d*)). At higher *Q* (greater than 1 Å^−2^) the data fits well to the Chudley–Elliot jump (CE) diffusion model. The *Q* values at 1.66 and 1.72 Å^−1^ are excluded due to potential contribution from Bragg peaks. The data were collected on an IRIS instrument and were grouped over 17 detectors.

The QENS data for the zeolite denoted as ZSM-5-40 are depicted in figure 3.

The nature of methanol mobility is analysed by the EISF and compared with different theoretical models in figure 3*b*. The experimental EISF is compared with effective EISF models in figure 3*c*, and the experimental data at low *Q* are described well by the translational diffusion model of methanol in a confined pore volume with an estimated radius of 3.36 Å and with a mobile fraction of around 35%. Note that methanol diffusion dynamics have switched from rotational to translational upon increasing the ratio from 11 to 40, which holds true for ZSM-5 with Si/Al ratios of 25 [25] and 36 [24], indicating the crucial role of the *Brønsted* acid site density in the methanol diffusion dynamics. It is noteworthy that among the zeolites with the Si/Al ratios of 25, 36 and 40, the confined pore radius is increased from 2.75 Å for ZSM-5-25 and ZSM-5-36 to 3.36 Å for ZSM-5-40 [24,25], indicating a greater methanol mobility at intersections upon reducing the acid site density of the zeolite [27]. The HWHM of the Lorentzian function appear to be *Q*^2^ independent between 0.2 and less than 1 Å^−2^ and *Q*^2^ dependent above 1 Å^−2^ as shown in figure 3*d*. The former is consistent with the VD model while the latter fits to the CE jump diffusion model. According to the VD model, diffusion of methanol is localized confined within a sphere and hence HWHM values are *Q*^2^ independent up to 1 (Å^−2^), which corresponds to the pore diameter of the confinement and the energy corresponds to the self-diffusion coefficient (*D*_s_) [24,25,30]. The *Q*^2^ of 0.9 (Å^−2^) corresponds to the estimated pore radius of 3.36 Å by EISF fit and matches with the radius of an intersection of H-ZSM-5 (i.e. 3.6 Å) [24]. The VD model results in a methanol *D*_s_ of 8.5 × 10^−10^ m^2^ s^−1^ and the CE jump diffusion fit gives a *D*_s_ of 8.3 × 10^−10^ m^2^ s^−1^ with a jump distance of around 2.2 Å and a residence time (*τ*) of 9.7 ps (table 2).

**Table 2.** Self-diffusion coefficient and the corresponding jump diffusion parameters are derived by Volino–Dianoux (VD) and Chudley–Elliot (CE) models. The QENS data obtained at 373 K over ZSM-5 with varied Si/Al.

			self-diffusion coefficient (*D*_s_), m^2^ s^−1^	
Si/Al	jump distance (*d*), Å	residence time (*τ*), ps	VD	CE
40	2.2	9.7	8.5 × 10^−10^	8.3 × 10^−10^
140	3.5	11.5	—	1.6 × 10^−9^

#### (iii) H-ZSM-5 with Si/Al of 140

**Figure 4.**
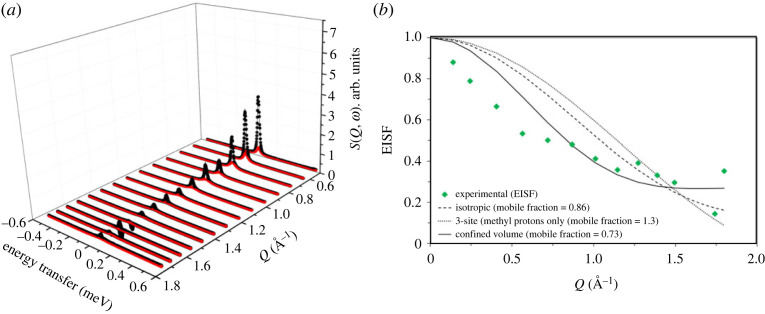
(*a*) QENS spectra of methanol loaded H-ZSM-5 with Si/Al of 140 at 373 K. Experimental data (filled circles), total fit (intense feature shown as a solid black line in the colour version online) and the Lorentzian function (broader, lower intensity function shown in red in the colour version). (*b*) The corresponding EISF (green diamond symbols) are modelled by including a fraction of immobile methanol in different theoretical models [12,16]. *Q* values at 1.58 and 1.67 Å^−1^ are not considered in (*b*) due to potential contribution from Bragg peaks. The data were collected on an OSIRIS instrument and were grouped over 14 detectors.

The QENS data for this zeolite, labelled as ZSM-5-140, are shown in figure 4.

**Figure 5.**
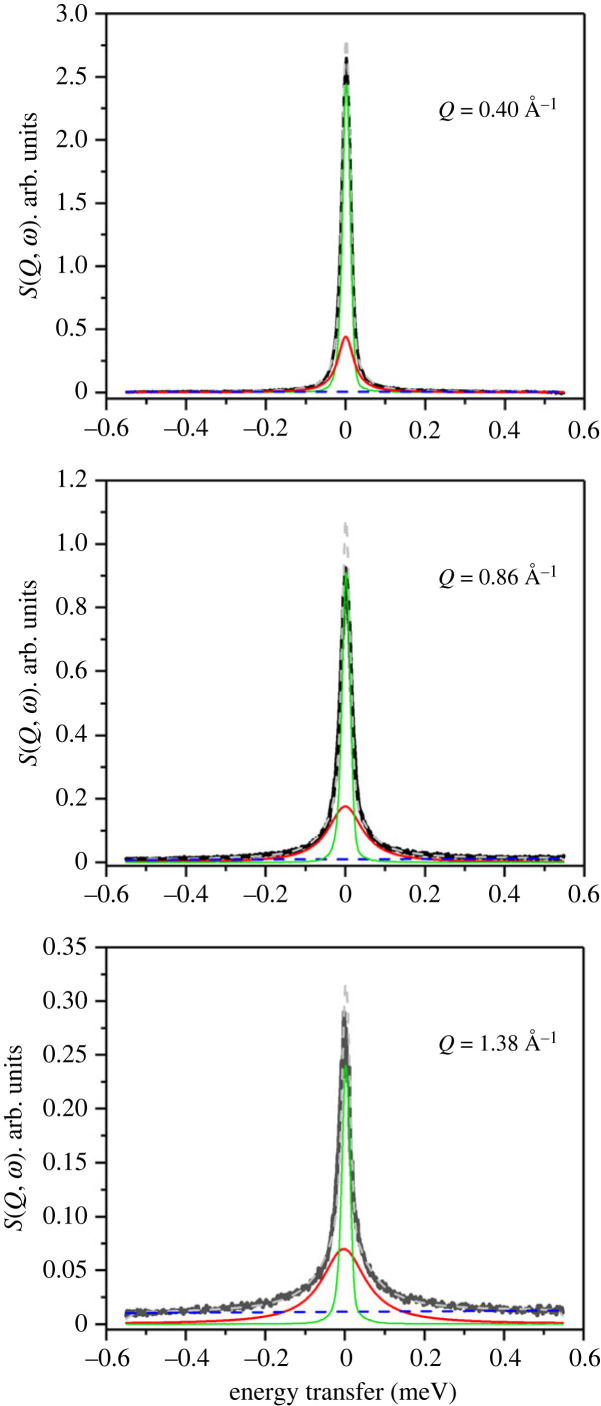
Selected QENS spectra of methanol loaded ZSM-5-140 measured at 373 K. The components are described in decreasing peak height; total fit (grey dashed line), experimental data (black solid line), resolution function (green solid line), the Lorentzian function (red solid line) and the linear background function (blue dashed line).

The QENS peak intensity at *Q* ≈ 0.6 (Å^−1^) is around 57% and 25% lower for ZSM-5-140 than that for ZSM-5-11 and ZSM-5-40, respectively. The absolute methanol uptake by the zeolites is dependent on the Si/Al ratio and hence the peak intensities are, in general, consistent with methanol uptake by the zeolites. Also, the drop in intensity of the elastic component of the QENS spectrum with increasing *Q* (Å^−1^) is more dramatic for ZSM-5-140 than those for ZSM-5-11 and ZSM-5-40. The effective EISF models fail to describe the experimental data. It can be clearly seen that the EISF decay is rapid below 1 (*Q*, Å^−1^), which would suggest long-range translational methanol diffusion [12]. Accurate assessment of this is limited by determination of the elastic component due to the instrumental resolution. This observation is in marked contrast to the one reported above for ZSM-5 with Si/Al ratios of 11 and 40 and also reported previously for ratios of 25 [25], 30 [16] and 36 [24]. In line with these observations, the QENS peak shape fits to the single Lorentzian function, whose width becomes prominent with increasing *Q* (figure 5).

The single Lorentzian function indicates the presence of a single type of methanol mobility, within the dynamic range of the instrument, in zeolite ZSM-5-140 at 373 K. The increase in peak width at lower *Q* between 0.4 and 1.1 Å^−1^ implies the occurrence of long-range translational methanol diffusional dynamics, in agreement with EISF data fit that does not fit to any localized methanol models (figure 4*b*). The broadening of the inelastic peak as a function of *Q*, as measured by the HWHM of the Lorentzian function, in figures 4*a* and 5 is clear evidence of dynamical behaviour of the sorbed methanol.

**Figure 6.**
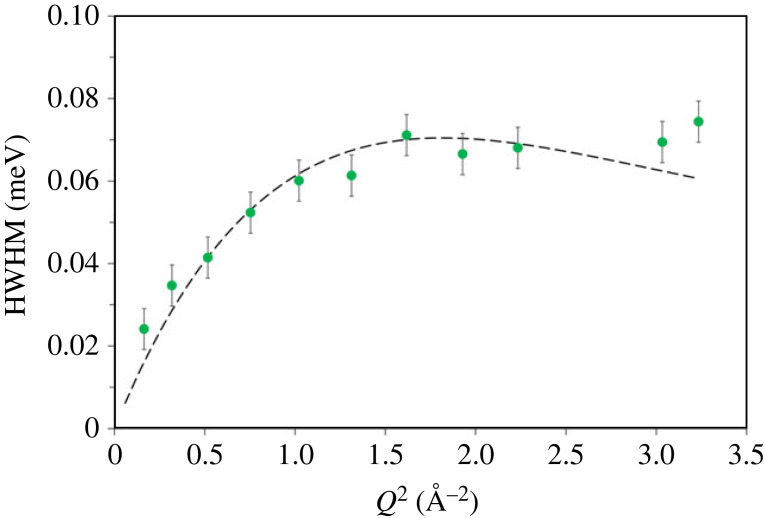
The *Q* dependence of the HWHM of the Lorentzian function (circles) fitted to the Chudley and Elliott jump diffusion model. The HWHM values at *Q* of 0.24, 1.58 and 1.67 Å^−1^ are not considered. Methanol motions at the lowest *Q* of 0.24 Å^−1^ are too slow to be accurately determined by the OSIRIS instrument under these experimental conditions.

The resulting HWHM of the Lorentzian functions is plotted as a function of *Q*^2^ in figure 6 and it increases sharply with *Q*^2^ from 0.4 to above 1.3 Å^−2^ and thereafter stabilizes. The CE jump diffusion model describes the data very well as is evident from the HWHM values that follow the CE model well in figure 6 [12]. The predicted jump distance, residence time and self-diffusion coefficient are presented in table 2.

Based on this analysis, we suggest that the methanol molecules jump from one adsorption site to another through the zeolite pores and the relative distance between the two sites is estimated to be around 3.5 Å, which is within the ZSM-5 pore size. Between the subsequent jumps, the methanol residence time on a site is calculated to be approximately 11.5 ps. A sufficient number of jumps through the zeolite pores results in a methanol diffusion coefficient (*D*_s_) of around 1.6 × 10^−9^ m^2^ s^−1^.

The *D*_s_ value is almost an order of magnitude higher than that reported previously for methanol diffusion in ZSM-5 with an Si/Al ratio of around 30 [12], which can be attributed to the differences in the Si/Al ratio of the zeolites, measurement temperature (the present study at 373 K versus the previous study at 300 K) and instruments with slightly different time and energy resolutions employed [12,24,25]. Given the higher measurement temperature and higher Si/Al ratio of the zeolite in the present study as compared with the previous report [12], the observed higher *D*_s_ value is not surprising. Moreover, translational diffusion of methanol is observed in zeolite H-Y (Si/Al = 30) with a larger pore size than the zeolite H-ZSM-5 using the OSIRIS instrument, suggesting that under reduced confinement, methanol mobility is quicker [35]. The methanol mobility (at 360 K) in zeolite H-Y is determined to be Fickian diffusion with a diffusion coefficient of approximately 4 × 10^−10^ m^2^ s^−1^ [35]. Given the time scale measured for zeolite H-Y, it can be concluded that the above observed *D*_s_ values of either 8.3 × 10^−10^ (as discussed above for figure 3*d*) or 1.6 × 10^−9^ m^2^ s^−1^ (figure 6 and table 2) indicate the intrinsic nature of methanol diffusion in ZSM-5 pores rather than the instrumental resolution of the spectrometers employed for this study. The difference in the *D*_s_ values determined for ZSM-5-40 (figure 3*d*) and ZSM-5-140 (figure 6) is indeed consistent with the Si/Al ratio of the zeolites; the higher the acid site density, the slower the diffusion rate.

It is evident from the above QENS results that the Si/Al ratio of the zeolite determines the methanol mobility and the nature of methanol dynamics. The highest acid site density zeolite ZSM-5-11 shows isotropic rotation of methanol with a mobile fraction of around 22%. By reducing the acid site density in ZSM-5-40, the mobile fraction increases (35%) and the dynamics switch from rotational to translational that is confined to a pore volume; similar methanol dynamics are observed in zeolites with Si/Al of 25 and 36 [24,25]. After further significant reduction in the acid site density in ZSM-5-140, the methanol mobility increases dramatically (figures 4–6) and translational diffusion of methanol is no longer confined to a pore. The results suggest that the higher the Si/Al ratio, i.e. the lower the acid site density, the greater the methanol mobility in ZSM-5 pores [24,25]. Accordingly, the confinement of methanol appears to be significantly reduced in ZSM-5-140, which leads to facile methanol mobility between the pores of the zeolite rather than localized motion within a pore as observed in ZSM-5-40. The key role of the Si/Al ratio of the zeolite in methanol mobility and diffusional dynamics is further assessed by the QENS data collected at less than 323 K [25].

### (b) Methanol dynamics at less than or equal to 323 K

**Figure 7.**
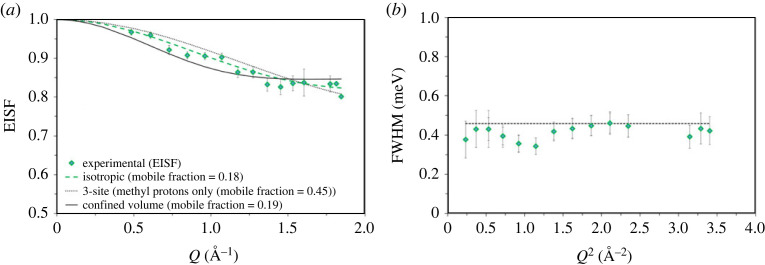
(*a*) Experimental EISF data of methanol loaded ZSM-5-11 at 323 K are modelled by including a fraction of immobile methanol in different theoretical models. Mobile methanol fraction is shown in the parenthesis of (*a*). (*b*) The FWHM of the Lorentzian function is plotted as a function of *Q*^2^. The potential contribution from Bragg peaks appears at *Q* values of 1.60, 1.66 and 1.72 Å^−1^ and hence values at 1.66 and 1.72 are excluded in (*a*). The corresponding FWHM values at 2.57, 2.78 and 2.97 *Q*^2^ are discounted in (*b*). The data were collected on an IRIS instrument and were grouped over 17 detectors.

The QENS data collected between 298 and 323 K over zeolites with varied Si/Al ratios of 11, 25 [25], 30 [16] and 36 [24] show isotropic rotation of methanol, which is different from the dynamics observed at 373 K (see §5a), except for ZSM-5-11, indicating that methanol diffusion dynamics in ZSM-5 pores are temperature dependent. Thus, less than or equal to 323 K, where isotropic rotation of methanol occurs in zeolites independent of Si/Al ratios, enables us to assess the effect of Si/Al ratio of ZSM-5 on the methanol mobility and rotational dynamics. As an example, the QENS data collected over ZSM-5-11 at 323 K are presented in figure 7.

**Figure 8.**
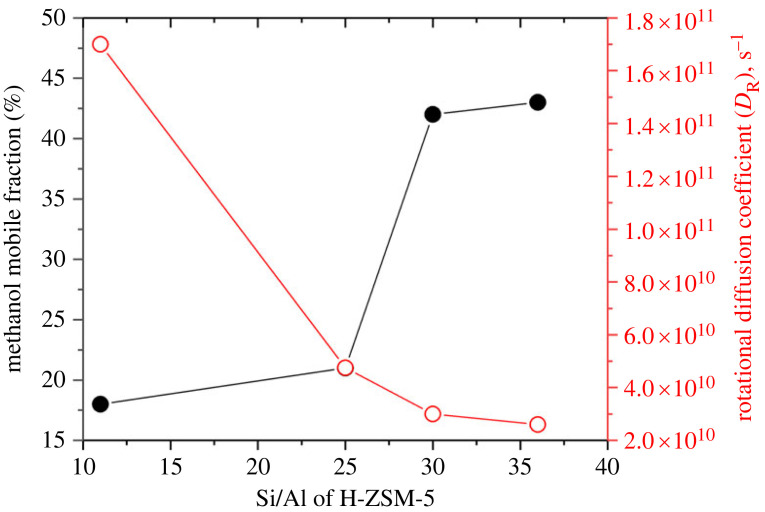
Correlation between methanol mobile fraction (%) (open symbols) and rotational diffusion coefficient (*D*_R_) (filled symbols) as a function of the Si/Al ratio of the zeolite H-ZSM-5. The correlation is derived from the present and previous studies reported in [16,24,25]. The methanol loading was at saturation level and QENS measurements were conducted between at 293 [24], 298 [25] and ≈323 K (present study and [16]).

The experimental EISF fits well to the isotropic methanol rotation model with a mobile fraction of 18%, which is around 18% lower than that observed at 373 K over the same zeolite in figure 1, indicating the lower the measurement temperature the lower the methanol mobility, as expected. The mobile fraction is around 15% lower than that reported for H-ZSM-5 with Si/Al of 25 (ZSM-5-25) at 298 K and is consistent with the acid site density of the zeolites. In line with the fit, the FWHM of the Lorentzian function appears to be *Q*^2^ independent, implying localized isotropic rotation of methanol. The FWHM give rise to a *D*_R_ of 1.73 × 10^11^ s^−1^, which is higher than that previously reported for ZSM-5-25 and is attributed to the Si/Al ratio of the zeolites [25]. Based on such studies, a correlation between the methanol mobile fraction (%) and rotational diffusion coefficient (*D*_R_) as a function of the Si/Al ratio is derived (figure 8).

It is evident that the mobile fraction increases and *D*_R_ decreases with increasing Si/Al ratio of H-ZSM-5 from 11 to 36, which is attributed to the acid site density of the zeolites; the higher the acid site density (i.e. lower the Si/Al ratio), the lower the methanol mobile fraction. The immobile methanol fraction is attributed to the occurrence of hydrogen bonded methanol and methoxy species at room temperature (RT), at which methanol loading is conducted for QENS experiments, as evident from infrared [36,37] and INS [15], indicating that methanol strongly interacts even at RT with acidic hydroxyls, especially with *Brønsted* acid sites, which are more reactive towards methanol than other hydroxyls [36–39]. It again signifies the important role of the Si/Al ratio in methanol dynamics. The methanol reactivity experiments are also complemented to some degree by QM/MM calculations, which show the occurrence of hydrogen bonded methanol at RT, but not methoxylation. The calculations also show that the structure (either neutral or protonated) of hydrogen bonded methanol is methanol loading dependent [40], consistent with infrared experiments [36–39].

The comparison between ZSM-5 with Si/Al ratios of 25 and 36 and that with 30 should be carefully considered due to dealumination of ZSM-5-30 during the MTH reaction leading to the formation of a fraction of mesopores and pore plugging. Note that ZSM-5 with Si/Al ratios of 25 and 36 are fresh zeolites and pore size distribution is experimentally determined. Nonetheless, the comparison presents a clear composition-dependent methanol mobility in zeolite H-ZSM-5 pores. There are slight variations in the experimental conditions and instrumental resolutions across the literature [12–16,25], requiring assessment of the experimental results by simulations, for which the classical MD simulations, which essentially operate over the same time and length scale resolutions of QENS, are very appropriate.

### (c) Classical molecular dynamics simulations

A recent study employed MD simulations to model methanol diffusion dynamics in zeolite H-ZSM-5 as a function of methanol loading and the Si/Al ratio of the zeolite [23]. The Si/Al ratios of 15, 47, 95, 191 and siliceous MFI, and methanol loadings of 3 and 5 molecules per unit cell (mpuc), were employed. The dynamics were studied at 373, 423 and 473 K. It is noteworthy that the Si/Al ratio of the zeolites and measurement temperature of 373 K are similar to our QENS data (present study and [23,24]), and that the measurement temperatures below 373 K have not been reported by MD. Another study reports the influence of zeolite topology (MFI and Beta) and acidity on the methanol diffusion [22]. For the purposes of comparison with our QENS experiments (§5a), we consider the measurement temperature of 373 K and methanol loading of five molecules per unit cell, which represents, to some degree, the experimental methanol loading at a saturation level (§5a,b).

**Figure 9.**
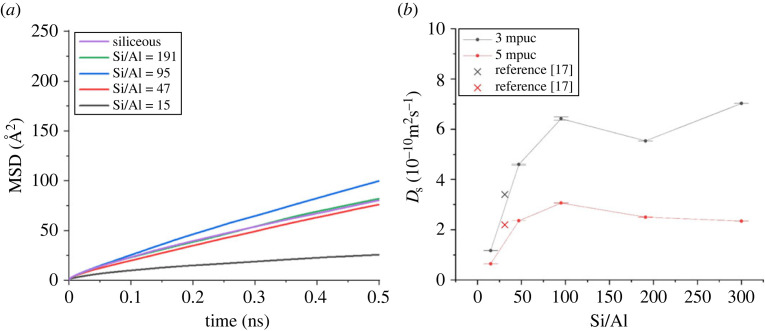
The mean-squared displacement (MSD) of methanol (5 molecules per unit cell; mpuc) in ZSM-5 pores with varied Si/Al ratios and in siliceous MFI pores (*a*) and the *D*_s_ of methanol in ZSM-5 pores as a function of the Si/Al ratio (*b*). Siliceous MFI is represented by Si/Al of 300 for clarity. The figures are adapted from [23]. In (*b*), 5 mpuc is considered for this study and [17] refers to [22].

The diffusivity of the methanol molecules was determined by tracking the positions of the carbon atoms of each methanol molecule and then the mean-squared displacements (MSD), self-diffusion coefficients (*D*_s_) and activation energies (*E*_a_) of diffusion were calculated [22,23]. The corresponding MSD and *D*_s_ are shown in figure 9 and *D*_s_ and *E*_a_ values are listed in table 3.

**Table 3.** Self-diffusion coefficients and activation energies derived by MD simulations for ZSM-5 with varied Si/Al ratios at 373 K. Methanol loading of 5 mpuc.

Si/Al	*D*_s_ (m^2^ s^−1^)	*E*_a_ (kJ mol^−1^)
15	0.65 × 10^−10^	10.4 ± 0.059
31	2.2 × 10^−10^	13.0
47	2.36 × 10^−10^	7.23 ± 0.017
95	3.06 × 10^−10^	7.98 ± 0.045
191	2.5 × 10^−10^	8.14 ± 0.03
siliceous	2.34 × 10^−10^	10.67 ± 0.112

The MD simulations in this study only sample the longer time range motions, i.e. the nanoscale diffusion through the zeolite framework—rather than the localized motions, and do not specifically take into account the instrumental resolution and time window (recently carried out for methanol in FER catalysts [27]), so observe translational diffusion of methanol with 100% mobility in ZSM-5 irrespective of the Si/Al ratio (15, 47, 95, 191 and siliceous MFI). It is evident from figure 9 that the MSD and *D*_s_ increase with increasing Si/Al from 15 to 95 and a dependence on composition is not seen at higher ratios (figure 9). The zeolite with the highest acid site density (Si/Al = 15) shows the least methanol diffusivity followed by zeolite with ratios of 31 and 47, consistent with the acid site density of the zeolite. The predicted methanol diffusivity in zeolites with Si/Al of 15 and 47 is relatively low and it complements the QENS data that show no *D*_s_ of methanol in zeolites with Si/Al of 11 (present study), 25 [25], 30 [16], 36 [24] and 40 (present study). MD simulations predict 100% methanol mobility in zeolites [22,23,27], while QENS shows that the majority of methanol is immobile in the zeolites; e.g. up to 80% methanol is immobile in ZSM-5-11, as reported in figure 1. The dichotomy of the results derived by the two techniques is attributed to the time resolutions of the techniques and emphasizes the importance of employing complementary techniques with identical time resolutions to obtain comprehensive information of a complex reaction like methanol diffusion dynamics in ZSM-5 pores. Clearly, the relatively slower methanol diffusivity in the above noted zeolites could not be detected by OSIRIS and IRIS instruments at ISIS as discussed for ZSM-5-140. In particular, the presence of dominant immobile methanol results in an intense elastic peak that dominates the quasi-elastic broadening, which might be lost in the background. This assumption gathers strength by the fact that the zeolite with Si/Al of 140 shows a significant quasi-elastic component (on the same instrument) when the majority of methanol is mobile (86% in ZSM-5-140), which minimizes the elastic peak contribution to a great extent, as discussed in §5a (figure 6). In line with this, the higher resolution instrument IN10 at ILL measures *D*_s_ of methanol of 10^−11^ m^2^ s^−1^ in ZSM-5 with Si/Al of 30, while the lower resolution instrument IN6 (≈1–41 ps) at ILL detects only localized methanol in the zeolite [12]. By contrast, the intermediate resolution of OSIRIS and IRIS instruments is capable of picking up both the localized and non-localized methanol motions under certain conditions, as demonstrated in §2a,b [15,16,24,25,35]. It is noteworthy that the *D*_s_ (≈10^−11^ m^2^ s^−1^) of methanol derived by the IN10 instrument is close to the simulated value (≈2.2 × 10^−10^ m^2^ s^−1^) for ZSM-5 with Si/Al of 31, which is comparable with the one employed for experiments at IN10, ILL [12].

Simulations also predict that zeolites with an Si/Al ratio of greater than or equal to 95, which have less than one *Brønsted* acid site per unit cell, and siliceous MFI show comparable methanol mobility and *D*_s_ [23]. Although no experimental data are available to verify this interesting prediction, it is assumed that the effect of acid site density on the methanol diffusion dynamics is almost diminished at Si/Al ratios of greater than or equal to 95 and only the pore architecture determines the dynamics at and above this ratio. The pore architecture could be one of the potential factors for calculated diffusivities as simulations show that methanol mobility and diffusivity are slower in siliceous MFI than that in siliceous Beta, which differ significantly in their topology and porous architecture [22], which is in line with MD simulations that show sorbate size-dependent diffusivity; maximum diffusivity is observed when the size of a sorbate is close to the size of the sorbent pore size [41]. We also note that there appear to be slight variations in the simulated values of *D*_s_ for similar zeolites. For instance, the *D*_s_ of methanol in siliceous MFI with 5 mpuc loading and at 373 K varied between 4.5 × 10^−10^ m^2^ s^−1^ and 2.3 × 10^−10^ m^2^ s^−1^ [22,23]. The variation might originate from the construction of models, which again points to the sensitivity of methanol dynamics to the local environment in the zeolite pores. The *E*_a_ for methanol diffusion (*D*_s_) appears to be higher for zeolite with Si/Al of 15 and it decreases for zeolite with the ratio of 47. Above this ratio the *E*_a_ begins to increase for zeolites with ratios of 95 and 191, and siliceous MFI presents the highest *E*_a_ (table 3). Subsequently, the highest acid site density zeolite ZSM-5-15 and siliceous MFI exhibit almost identical values of *E*_a_ of 10.5 kJ mol^−1^ which is intriguing and requires experimental verification. However, on decreasing the methanol loading from 5 to 3 mpuc, a clear trend appears in *E*_a_, which decreases with increasing Si/Al [23]. Again, no experimental evidence is currently available to verify these simulations. The above results highlight the complexity and sensitivity of methanol dynamics in ZSM-5 pores.

## 6. Summary and outlook

Methanol diffusion dynamics in zeolite ZSM-5 pores have been evaluated by QENS and MD simulations. Critical analyses of the current and previous data reveal that the Si/Al ratio of the zeolite and measurement temperature determine the methanol diffusion dynamics in ZSM-5 pores. It is clear from QENS data that the higher the *Brønsted* acid site density (i.e. lower the Si/Al ratio), the lower the methanol mobility. The nature of methanol diffusion also depends on the acid site density and measurement temperature. The former is illustrated in §5a by zeolites with Si/Al ratios of 11, 25, 36, 40 and 140, and the latter is discussed in §5b and reported elsewhere using ZSM-5 with the Si/Al ratio of 25 [25]. Essentially, most of the methanol (up to 78% in ZSM-5-11) is immobile in high acid site density zeolites and the remaining mobile methanol fraction exhibits isotropic rotation (figure 1), detected within the instrumental resolution. As the acid site density decreases, the mobile methanol fraction increases (up to 86% in ZSM-5-140) and shows (depending on the Si/Al) either confined diffusion within a pore volume (e.g. in intermediate Si/Al of 40, figure 3) or long-range translational diffusion (without confinement) across the pores in ZSM-5-140 (figure 6). The immobile methanol fraction is attributed to the occurrence of both hydrogen bonded methanol and methoxy species at room temperature, as evident from infrared spectroscopy [36–39], in line with previous work that concludes that Brønsted acid sites are the active sites for the conversion of methanol into hydrocarbons [1–4,6,8,11,15,36–40]. Accordingly, both the adsorption at the Brønsted acid sites, and the additional intermolecular interactions of methanol molecules leading to the formation of methanol clusters [36–40], restrict the methanol mobility in the zeolite pores.

MD simulations over longer time scales (with diffusion coefficients calculated over 1 ns) predict translational diffusion of methanol with 100% methanol mobility irrespective of Si/Al ratio (15, 47, 95, 191 and siliceous MFI) of ZSM-5 complementing the QENS data by predicting the occurrence of translational diffusion of methanol in high acid site density zeolite like in ZSM-15 [22,23,27]. However, there appears to be a dichotomy on the methanol mobile fraction derived by the two techniques. For instance, QENS shows only around 18% mobile fraction in ZSM-5-11 (figure 7), which has a Si/Al ratio comparable with that of ZSM-5-15, with a predicted 100% methanol mobility by MD simulations. These discrepancies are attributed to the time resolutions of the techniques employed [22,23,27].

Based on QENS and MD simulations, it is evident that different methanol diffusion dynamics occur at the same time in ZSM-5 pores and it appears to be difficult to detect all the possible dynamics at once by any technique. One potential way to detect different methanol dynamics is to employ complementary techniques. For example, QENS instruments with suitable energy resolutions to detect high energy methanol rotational and low energy methanol translational diffusion, as demonstrated by Jobic *et al*. [12] in ZSM-5-30 (Si/Al of ≈30) pores. However, even such an approach may not differentiate the translational diffusion of different hydrogenous species (including methanol and water, which is a by-product of methoxylation) that occur in zeolite pores because the width of the quasi-elastic peak is the result of all the species present in the pores. The latter holds true especially at higher temperature where reaction kicks in and product formation takes place. This raises the following questions and possibilities for future studies: (i) Can we distinguish dynamics of different species present in the pores? (ii) What is the role/effect of products (or by-products) on the methanol diffusion dynamics? and (iii) Can we conduct diffusion studies in real time under reaction conditions (i.e. operando methodology [42]). A productive approach would potentially begin with MD simulations to obtain basic information that then need to be corroborated by complementary experiments using QENS with suitable time and energy resolutions and other techniques as required. From a fundamental point of view, methanol diffusion dynamics in ZSM-5 pores are complex and it requires a multi-technique approach in order to obtain a clear and comprehensive understanding of the system, which may help to develop efficient processes either by zeolite or by reactor design.

## Data Availability

The raw experimental data resulting from our neutron beamtime RB1920441 can be found at doi:10.5286/ISIS.E.RB1920441 [43].
